# Influence of in vivo Hsp90α inhibition on irisin signaling to the brain

**DOI:** 10.1002/alz.091959

**Published:** 2025-01-09

**Authors:** Zachary J White, Keshari Sudasinghe, Ramona Weber, Stephanie E Hall

**Affiliations:** ^1^ Kansas State University, Manhattan, KS USA

## Abstract

**Background:**

Irisin is an exercise‐induced myokine that elicits beneficial effects of exercise in fat, bone, and the brain. Previous work suggests that extracellular heat shock protein 90a (Hsp90a) mediates irisin‐receptor interaction in bone and fat. Despite this, it remains unclear if Hsp90a is necessary for irisin signaling in the brain.

**Methods:**

Female F344 rats (3‐4 mo, n = 32) were randomized into four treatment groups: irisin + Hsp90a inhibition (IX, n = 10); irisin + vehicle o.g. (IC, n = 10); vehicle i.p. + Hsp90a inhibition (CX, n = 6) and; vehicle i.p. + vehicle o.g. (CC, n = 6). Due to the impact of irisin on memory, a novel object recognition test was conducted over 4 days. Days 1‐2 were habituation, day 3 was familiarization, and day 4 was testing. Directly following NOR day 2, animals received single administrations of irisin (100 mg/kg, i.p.) or vehicle control and selective Hsp90a inhibitor SNX‐5422 (10 mg/kg, o.g.) or vehicle control. Day 4 NOR testing and tissue collection occurred 48 hours following drug administrations. Blood was collected via LV puncture, centrifuged to isolate serum, and stored at ‐80°C. Brains were sectioned, weighed, and snap‐frozen in liquid nitrogen. The downstream target of irisin signaling, hippocampal cyclic‐AMP Response Element Binding Protein (CREB) was quantified via Jess Simple Western automated western blot analysis. Hippocampal BDNF and serum irisin were quantified via ELISA. Values are mean ± standard deviation.

**Results:**

In the NOR task, the IC group displayed a preference for the novel object (68.77 ± 17.93% of exploration time with novel object; p<0.01, one‐sample t‐test vs 50%). Novel object preference was not displayed in other groups. Hippocampal total CREB was greater in the IC than in the CC group (11.0 ± 0.85 vs 9.4 ± 0.80 a.u.; p<0.05, one‐way ANOVA). No differences in tissue masses, levels of hippocampal phosphorylated CREB, p/t CREB, and BDNF or serum irisin were present between groups 48 hours following dosing.

**Conclusions:**

This investigation supports irisin signaling to promote memory function and indicates a mediating role of Hsp90a in the brain. Further analysis of hippocampal mRNA is needed to understand the influence of Hsp90a inhibition on irisin‐mediated transcriptional changes.

